# Case Report: Synthetic mesh failure and biological mesh salvage in major oncologic abdominal wall reconstruction—a two-case series with technical considerations

**DOI:** 10.3389/fsurg.2026.1936273

**Published:** 2026-10-20

**Authors:** Ismail Saeed, Khalid Osama Alnajjar, Ali H. Alfuraydi, Ayman Zaki Azzam, Ihssan Alalem

**Affiliations:** 1 College of Medicine, Alfaisal University, Riyadh, Saudi Arabia; 2 Surgical Oncology Department, Cancer Centre of Excellence, King Faisal Specialist Hospital & Research Centre, Riyadh, Saudi Arabia; 3 General Surgery Department, Alexandria University, Alexandria, Egypt

**Keywords:** abdominal wall reconstruction, biological mesh, case report, CRS, mesh complications, permacol, synthetic mesh

## Abstract

**Background and objectives:**

Abdominal wall reconstruction after major oncologic resection is challenging, particularly when the operative field has been compromised by radiation, extensive tissue loss, and, in some cases, intraperitoneal chemotherapy. Mesh choice is central to minimizing complications and achieving durable outcomes. This report describes the clinical course of two patients who underwent initial synthetic mesh reconstruction and subsequently required biological mesh salvage, both managed at a single tertiary referral center.

**Methods:**

Two patients who underwent anterior abdominal wall reconstruction following major oncologic resection were retrospectively reviewed. Both initially received synthetic mesh (expanded polytetrafluoroethylene [ePTFE, Gore-Tex®] in Case 1; polypropylene-based mesh in Case 2). After developing mesh-related complications, both underwent reoperation with biological mesh (Permacol™ Surgical Implant, Covidien) placed in an underlay position with primary fascial closure.

**Results:**

Both patients developed recurrent surgical site infections, mesh protrusion, and bowel adhesions that required multiple interventions following synthetic mesh placement. After reconstruction with biological mesh, infection resolved and abdominal wall integrity was restored, with no further mesh-related complications at 12 months of follow-up in each case.

**Conclusion:**

These cases show how synthetic mesh can fail in the compromised field of major oncologic abdominal wall reconstruction, and suggest that mesh choice should be matched to the extent and contamination risk of the resection, with biological mesh a reasonable option when the field is heavily compromised. The findings are hypothesis-generating and should not be generalized. Prospective studies are needed to establish mesh selection criteria in this setting.

## Introduction

Major oncologic resection of the anterior abdominal wall frequently requires wide excision and complex reconstruction under physiologically challenging conditions. Adjuncts such as intraoperative and neoadjuvant radiation, extensive resection or cytoreductive surgery (CRS), and, in selected cases, hyperthermic intraperitoneal chemotherapy (HIPEC), further compromise wound healing and increase the risk of mesh-related complications (1).

Mesh reinforcement is frequently required to restore abdominal wall integrity and reduce the risk of herniation, yet the choice of mesh type in these high-risk oncologic settings remains unsettled (2). Biological, biosynthetic, and synthetic meshes each carry different risk profiles, and no prospective comparative data exist to support definitive recommendations for this patient group (3).

We report two patients (Table 1) in whom abdominal wall reconstruction with synthetic mesh following major oncologic resection was complicated by chronic infection, progressive mesh-related morbidity, and, in one case, erosion into the femoral artery, necessitating explantation. Both were subsequently managed with biological mesh reconstruction, with favorable outcomes at one year. These cases are presented as descriptive, hypothesis-generating observations to inform mesh selection in this difficult surgical context.

**Table 1:** provides a comparative summary and clinical timeline for both cases.

Parameter	Case 1	Case 2
Age/Sex	46-year-old male	37-year-old male
Primary diagnosis	Right pubic bone chondrosarcoma with anterior abdominal wall invasion	Recurrent left inguinoscrotal liposarcoma
Index surgery date	April 2018	February 2016
Index mesh type	Synthetic ePTFE (Gore-Tex®)	Polypropylene-based: C-QUR TachShield (PP with Omega-3 coating, Atrium) + Surgipro monofilament polypropylene (Covidien)
Rationale for synthetic mesh	Clean field at index surgery; large bony and soft tissue defect requiring durable structural support	Clean field at index surgery; wide inguinal defect requiring structural reinforcement
Oncologic procedure	CRS + bidirectional HIPEC (IV ifosfamide; IP mitomycin and doxorubicin; intraoperative decision) + IORT	Wide excision + IORT + neoadjuvant radiation
Bowel resection performed?	No	No
Intraoperative contamination?	Controlled (appendectomy)	Controlled (scrotal/perineal field)
CDC wound class (index surgery)	Class II (Clean-contaminated)	Class II (Clean-contaminated)
First complication onset	August 2018 (∼4 months post-index)	December 2018 (∼34 months post-index)
Complication pattern	Chronic, progressive: mesh protrusion, recurrent SSI, bowel adhesions, septic mesh	Chronic, progressive: MRSA infection, sinus tract, ECF, femoral artery erosion (Sep 2023), osteomyelitis (Dec 2023), bowel obstruction
Interim mesh surgery	Feb 2023: elective right inguinal hernia repair with additional dual ePTFE mesh (bone-anchored)	Sep 2020: partial mesh removal; Feb 2022: sinus debridement
Notable complication	Septic mesh requiring multiple debridements; right iliac/femoral DVT; IVC filter	Mesh erosion into left femoral artery (Sep 2023) requiring emergency endovascular stent; pubic ramus osteomyelitis (Dec 2023)
Salvage surgery date	December 2024	October 2024
Biological mesh used	Permacol™ Surgical Implant (Covidien)	Permacol™ Surgical Implant (Covidien)
Mesh placement plane	Underlay (sublay)	Underlay (sublay)
Primary fascial closure?	Yes	Yes
Follow-up duration	12 months (to December 2025)	12 months (to October 2025)
Outcome at follow-up	No infection, no hernia recurrence, intact abdominal wall	No infection, no hernia recurrence, intact abdominal wall

SSI, Surgical site infection; ECF, Enterocutaneous fistula; IORT, Intraoperative radiation therapy; DVT, Deep vein thrombosis; IVC, Inferior vena cava; CDC, Centers for Disease Control and Prevention; MRSA, Methicillin-resistant Staphylococcus aureus; PP, Polypropylene.

## Patients & methods

This retrospective case series reports two patients who underwent major oncologic anterior abdominal wall resection and reconstruction at King Faisal Specialist Hospital & Research Centre (KFSH&RC), a tertiary cancer referral center in Riyadh, Saudi Arabia. Data were obtained from medical records, operative reports, and imaging. Both patients initially received synthetic mesh reconstruction (ePTFE in Case 1; polypropylene-based mesh in Case 2) and later required reoperation for mesh-related complications, followed by reconstruction with Permacol™ Surgical Implant (Covidien), a porcine-derived cross-linked collagen biological mesh, placed in an underlay position with primary fascial closure in both cases.

Wound contamination was graded using the Centers for Disease Control and Prevention (CDC) and National Healthcare Safety Network (NHSN) wound classification criteria (4). Both index procedures were classified as CDC Class II (clean-contaminated), reflecting the contemporaneous operative classification: in Case 1 based on the appendectomy (controlled entry into a colonized viscus), and in Case 2 based on the entry into the colonized scrotal and perineal field during tumor resection and reconstruction. Written informed consent was obtained from both patients for publication of clinical data and images. The requirement for ethical approval was waived by the King Faisal Specialist Hospital & Research Centre Institutional Review Board for this retrospective case report, in accordance with local regulations governing the reporting of routine clinical care.

## Results

### Case 1

A 46-year-old Saudi male presented with right pubic bone chondrosarcoma invading the anterior abdominal wall. He had a background of prior thromboembolic disease requiring inferior vena cava (IVC) filter placement, and had received intraoperative radiation therapy (IORT) at the index procedure, both of which are relevant to wound healing and reconstructive outcomes.

In April 2018, he underwent wide tumor excision, appendectomy, CRS, and bidirectional HIPEC, with anterior abdominal wall reconstruction using synthetic ePTFE (Gore-Tex®) mesh. Because the tumor was large and given its close proximity to nearby structures, it could not be removed *en bloc* and was resected in a piecemeal fashion, resulting in intraoperative peritoneal tumor spillage; in response to this intraoperative finding, bidirectional HIPEC (intravenous ifosfamide with intraperitoneal mitomycin and doxorubicin) was administered to reduce the risk of subsequent peritoneal seeding. Given the extent of bony and soft tissue resection and the clean operative field at the time of surgery, synthetic mesh was chosen for its mechanical durability and structural support. Concurrent appendectomy constituted controlled entry into a colonized viscus, and the wound was classified as CDC Class II (clean-contaminated). No bowel resection was performed.

Approximately four months after surgery (August 2018), the patient began developing recurrent complications including mesh protrusion, bowel adhesions, and surgical site infections. In February 2019, given ongoing wound breakdown and abdominal wall deficiency, he underwent exploratory laparotomy with reconstruction of the lower right abdominal wall using a left rectus abdominis muscle flap. This was complicated by surgical site infection and right distal iliac and femoral vein thrombosis, managed with anticoagulation and IVC filter placement. He was subsequently followed in clinic, with no major intervention required until February 2023, when he was admitted for an elective right inguinal hernia repair; given the prior bone and inguinal-ligament resection, the defect was reconstructed with additional dual-layer ePTFE (Gore-Tex®) mesh anchored with bone screws, with omental coverage and antibiotic irrigation. Wound breakdown followed this procedure, and he underwent abdominal wound exploration in July, September, and October 2023, with abscess evacuation and debridement at each admission. Throughout this period, the mesh remained septic, causing progressive functional impairment and significantly reduced quality of life.

In December 2024, approximately six and a half years after the index procedure, the patient was admitted electively for definitive abdominal wall reconstruction. Intraoperatively, extensive adhesiolysis was performed and the retained, infected ePTFE (Gore-Tex®) mesh from the prior reconstructions—encompassing the 2018 index implant and the 2023 dual-layer mesh placed in the right inguinal region—was excised (Figure 1). Two concurrent abdominal wall hernias were repaired using Permacol™ Surgical Implant (Covidien), a 16 × 20 cm porcine acellular dermal matrix, placed in an underlay position with primary fascial closure in all defects. Two closed-suction (Jackson-Pratt) drains were placed and were removed sequentially, the last by approximately postoperative day 13 at the time of discharge.

**Figure 1:**
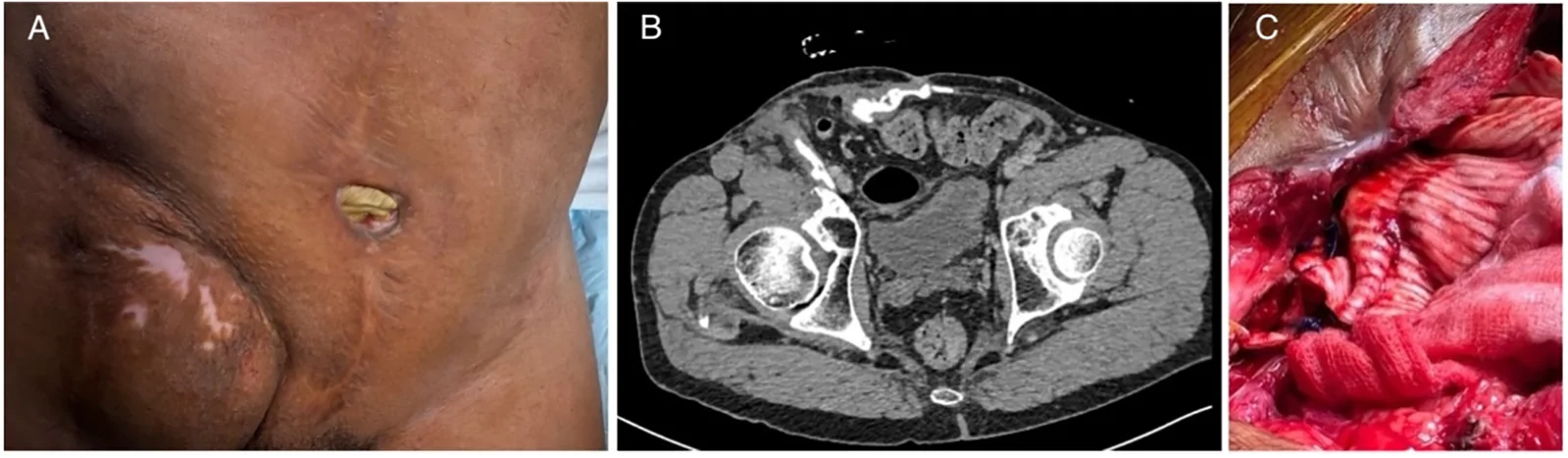
Case 1. **(A)** Extrusion of the synthetic Gore-Tex® mesh from the lower abdominal midline incision with surrounding signs of chronic infection and skin breakdown. **(B)** Axial CT image showing herniation of bowel loops through the anterior abdominal wall defect. **(C)** Intraoperative photograph of the explanted Gore-Tex® mesh with no visible tissue integration and macroscopic features of infection.

Postoperatively, the patient was vitally stable and afebrile. Abdominal examination showed a soft, lax abdomen with localized tenderness over the surgical site and no signs of peritonitis. The midline wound was clean and dry with no discharge or signs of infection. Two Jackson-Pratt drains were *in situ* with minimal serous output. At 12 months of follow-up (December 2025), the patient had achieved complete wound healing with no evidence of hernia recurrence or active infection (Figure 2).

**Figure 2:**
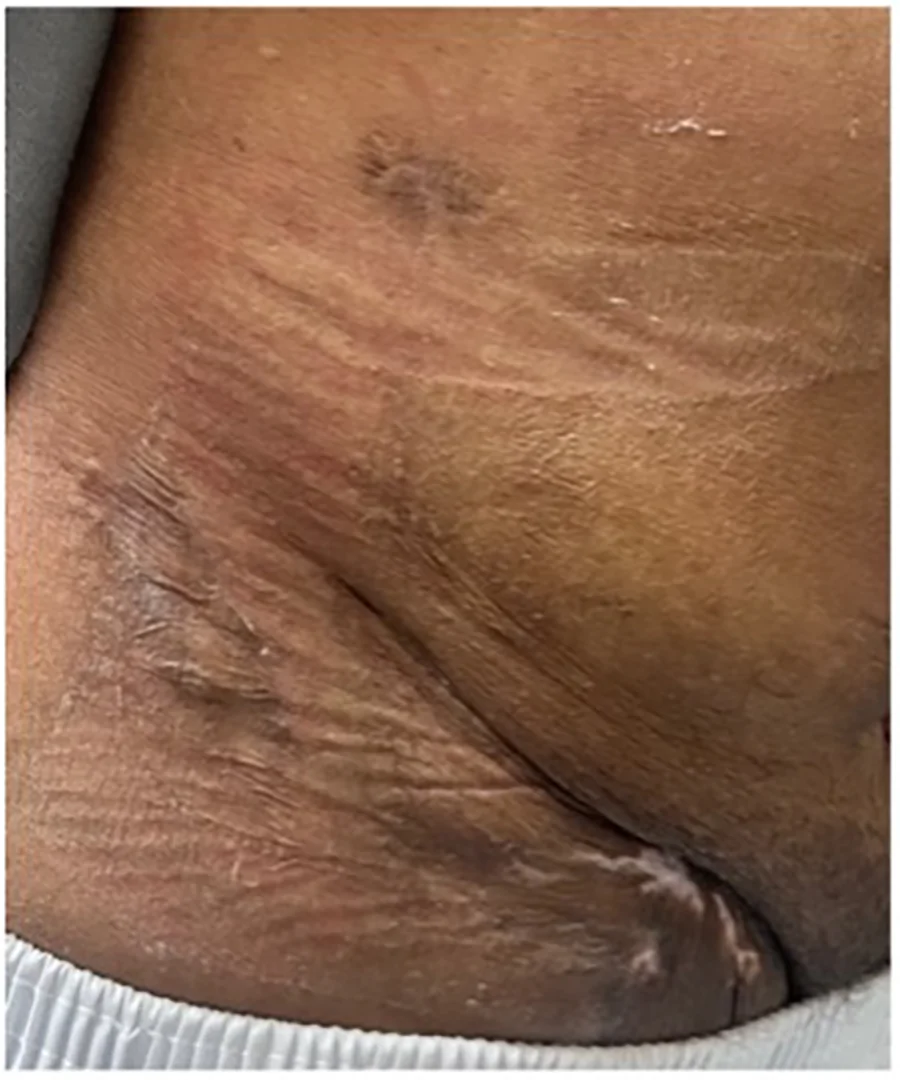
Case 1. Wound appearance at 12 months following biological mesh reconstruction, showing complete healing with no clinical evidence of hernia recurrence or active infection.

### Case 2

A 37-year-old Saudi male presented with recurrent left inguinoscrotal liposarcoma. He had a complex oncologic history with the primary diagnosis established in 2007. Relevant comorbidities included prior neoadjuvant radiation to the operative field, lower limb deep vein thrombosis managed with anticoagulation, and a prior episode of brucellosis. He had documented drug allergies to Tazocin and Bactrim, with reactions including urticaria, pruritus, and bronchospasm, which significantly limited antibiotic options throughout his subsequent clinical course.

In February 2016, following recurrence, he underwent wide tumor excision with IORT and anterior abdominal wall reconstruction using polypropylene-based synthetic mesh placed in two planes: a polypropylene mesh with an Omega-3 fatty acid bioabsorbable coating (C-QUR TachShield, Atrium) placed as a sublay to reconstitute the left rectus sheath and inguinal area, with monofilament polypropylene mesh (Surgipro, Covidien) placed as an overlay to reinforce the right side of the abdominal wall. Intraoperative radiation was delivered to the pubic tubercle and left inguinal region (12.5 Gy medially and 10 Gy laterally). Synthetic mesh was selected given the clean operative field and the structural demands of the wide inguinal defect. Entry into the colonized scrotal and perineal field during resection constituted controlled contamination, and the wound was classified as CDC Class II (clean-contaminated). No bowel resection was performed.

From December 2018, approximately 34 months postoperatively, the patient developed a pattern of progressive complications including recurrent mesh infections, mesh protrusion, bowel adhesions, and a malodorous sinus tract secondary to an enterocutaneous fistula (ECF) in the left groin (Figure 3A). Microbiological cultures confirmed methicillin-resistant Staphylococcus aureus (MRSA) as the causative organism; antibiotic selection was further constrained by his documented allergies to Tazocin and Bactrim, necessitating meropenem-based intravenous therapy across multiple admissions. Partial mesh removal was carried out in September 2020 for ongoing sinus infection, and he underwent further sinus debridement in February 2022; however, residual mesh remained *in situ* and continued to act as a nidus for recurrent infection.

**Figure 3:**
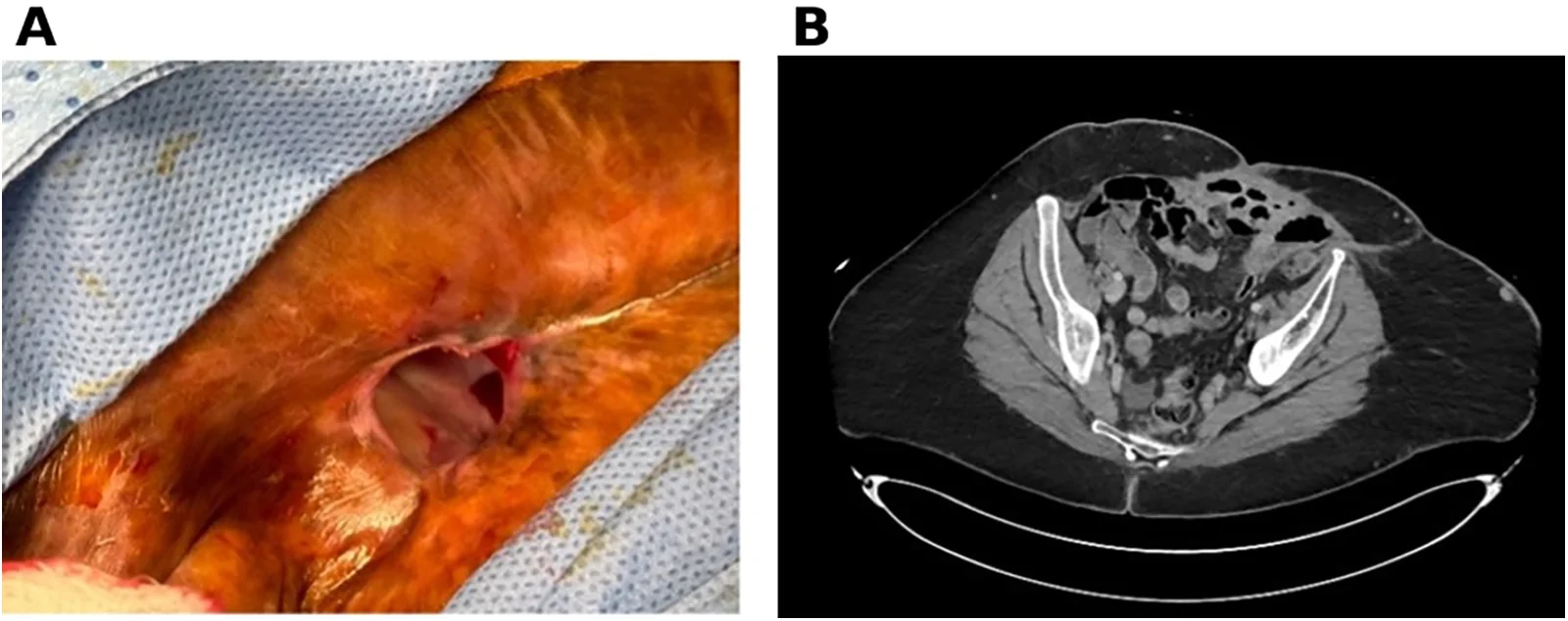
Case 2. **(A)** Intraoperative image showing an enterocutaneous fistula in the left groin with surrounding infection and sinus tract formation associated with chronic synthetic mesh infection. **(B)** Axial CT image showing extensive bowel adhesions and ongoing inflammatory change in the left lower quadrant surrounding the synthetic mesh.

In September 2023, the patient developed acute hemorrhage from the left groin. Intraoperatively, the synthetic mesh was found to have eroded directly into the wall of the left femoral artery; emergency endovascular stenting was performed, with the stent spanning the external iliac–common femoral segment, and the bleeding was controlled. The eroding mesh corresponded to the segment overlying the left femoral vessels in the inguinal reconstruction; only its peri-vascular portion was removed at this stage, as the remainder was incorporated within the reconstructed abdominal wall. Subsequent workup in December 2023 identified pubic ramus osteomyelitis as a further sequela of the chronic infectious process. Cross-sectional imaging confirmed progressive bowel adhesions (Figure 3B), and the patient's condition deteriorated further with ongoing infection and intermittent bowel obstruction.

On examination at the time of the 2024 admission, the patient presented with a malodorous left groin wound with active purulent discharge, a palpable sinus tract, and surrounding erythematous induration consistent with chronic mesh infection.

In October 2024, definitive salvage surgery was performed. Intraoperatively, the previously placed sublay mesh was found to be well integrated and without active infection; the residual synthetic mesh overlying the femoral vessels was identified as the focus of chronic infection and was excised under vascular control, with the previously placed stent left intact. The ECF was confirmed and addressed, and an iatrogenic bowel injury was managed with segmental bowel resection and primary anastomosis. The pelvic floor was then reconstructed with Permacol™ Surgical Implant (Covidien), a 15 × 20 cm porcine acellular dermal matrix, which was trimmed to the defect, positioned to cover the pelvic floor and prevent small-bowel herniation into the thigh, and secured anteriorly to the abdominal wall. Midline fascial closure was achieved with looped PDS sutures under no undue tension, and the previously raised myocutaneous flap was reutilized for soft-tissue coverage. Two drains (one abdominal, one pelvic) were placed; drain-removal timing was not documented, and the patient was discharged with a referral for home wound care. At 12 months of follow-up (October 2025), the patient had no evidence of hernia recurrence, no active infection, and intact abdominal wall integrity.

## Discussion

In both patients, synthetic mesh placed after major oncologic abdominal wall resection failed and was ultimately salvaged with biological mesh. These are illustrative observations; no causal conclusions can be drawn from two cases, and the findings should not be extrapolated beyond this context.

The improvement seen after biological mesh reconstruction cannot be attributed to the mesh alone. Both salvage procedures were carried out in a more favorable operative environment, after the infected synthetic material had been removed, antibiotic therapy completed, and the acute infection resolved. The effect of this improved context cannot easily be separated from any benefit of the biological mesh itself. A 2023 systematic review found no significant difference in recurrence rates between biological and synthetic meshes in clean-contaminated and contaminated fields when baseline conditions were controlled, which further underlines the role of operative context in interpreting mesh outcomes (3).

Abdominal wall reconstruction after major oncologic resection is demanding. Even without bowel entry, the combination of radiation exposure, extensive tissue resection, prolonged operative time, and impaired wound healing raises the risk of mesh-related complications well above that of elective hernia repair (1, 2). Both patients had received radiation to the operative field, which impairs healing and mesh tolerance; Case 1 additionally underwent CRS and HIPEC, a decision made for a rare presentation without established guidelines (5). In both, the field was classified as CDC Class II at index surgery, reflecting the oncologic resection rather than overt contamination. Despite this, both developed chronic, progressive mesh-related morbidity that required explantation.

Synthetic ePTFE meshes offer good mechanical strength and acceptable recurrence rates in clean fields (6, 7). Their main limitation in contaminated settings is that they do not incorporate into surrounding tissue and are prone to bacterial biofilm formation, which makes clearing infection without explantation unlikely (2). Laboratory studies show that ePTFE supports bacterial adherence at very low inoculum concentrations and achieves less neovascularization than biological alternatives, which may explain its behavior in Case 1 (8). In Case 2 the failed mesh was polypropylene-based, and the mechanism differed: chronic infection with multidrug-resistant organisms in an irradiated field, together with direct mechanical erosion of mesh lying against the femoral vessels, drove the morbidity. Even a coated polypropylene mesh intended to improve integration failed here, which points to the hostile oncologic field, rather than any single material, as the dominant factor. The initial choice of synthetic mesh in both patients was reasonable given the clean field at index surgery; in retrospect, the prolonged physiological insult of major oncologic resection and radiation may have undermined the conditions needed for long-term synthetic mesh tolerance.

Biological meshes, including porcine-derived collagen implants such as Permacol™, incorporate into host tissue through neovascularization and are better tolerated in infected or high-risk environments (9, 10). Favorable outcomes with biological mesh in oncologic abdominal wall reconstruction have been reported by Baglien et al. and Tzivanakis et al. (9, 11), consistent with what we observed. Biological mesh has limitations, however: recurrence rates are higher when it is used as a bridge without achieving primary fascial closure (12). Primary closure was achieved in both our patients, which likely contributed to the durability of the reconstruction at 12 months.

Biosynthetic meshes, made from degradable synthetic polymers such as polyglycolic acid and trimethylene carbonate, may offer a middle ground in moderately contaminated fields by combining infection resistance with structural support (13, 14). The coated polypropylene mesh used in Case 2 did not withstand the chronically infected, irradiated field, which suggests that partial material modifications may be insufficient in the most hostile oncologic settings. These meshes may still be worth evaluating in future comparative studies.

The femoral artery erosion in Case 2 merits comment. Chronic synthetic mesh infection in the groin, with mesh lying directly against the femoral vessels in a heavily irradiated field, set up the conditions for direct arterial wall erosion and life-threatening hemorrhage, confirmed intraoperatively. On a structured PubMed search, we found no prior report of synthetic mesh erosion into the femoral artery following oncologic abdominal wall reconstruction, so this appears to be the first such case. The erosion (September 2023) preceded the diagnosis of osteomyelitis (December 2023), which points to the mesh, rather than the osteomyelitis, as the primary driver of the vascular injury. Surgeons undertaking groin-based oncologic reconstruction should keep a low threshold for early mesh explantation when infection is suspected near major vessels.

Several limitations apply. The series is small, with no control group and no ability to perform statistical analysis. The retrospective design carries inherent bias, and we have no denominator to indicate how many similar patients tolerated synthetic mesh without complications. Microbiological data are incompletely reported for Case 1, which limits the extent to which the biofilm-based mechanism (8) can be applied to that case specifically. Follow-up of 12 months, while encouraging, is insufficient to exclude late hernia recurrence, which is known to increase over time with biological mesh (12). The substantial cost of biological mesh relative to synthetic alternatives was not formally analyzed but represents a practical constraint on its routine use (2). In addition, the specific suture used to fix the biological mesh was not recorded in the operative documentation available for retrospective review. Larger prospective studies with standardized outcome measures are needed to guide mesh selection in this population.

## Conclusion

These two cases show that synthetic mesh can fail progressively after major oncologic abdominal wall reconstruction even when the field is classified as clean-contaminated, and that biological mesh reconstruction in an underlay position with primary fascial closure restored abdominal wall integrity at one year. The femoral artery erosion in Case 2, alongside MRSA infection and pubic ramus osteomyelitis, is a rare and under-reported vascular complication of chronic synthetic mesh infection in the groin that surgeons managing similar patients should be aware of. Taken together, the cases suggest that mesh choice should be matched to the extent and contamination risk of the oncologic resection, with biological mesh worth considering in the most heavily compromised fields; they should be read as hypothesis-generating rather than practice-defining. Importantly, these cases do not establish that biological mesh should have been the index choice; synthetic mesh was a reasonable selection in the clean fields presented at the primary operations, and these observations speak to the role of biological mesh as salvage after synthetic mesh failure rather than as a first-line implant. Well-designed prospective studies are needed to establish mesh selection criteria in this population.

## Patient perspective

Case 1: The patient described the years of repeated infections, hospital admissions, and wound dressing changes as having a significant impact on his daily life and ability to work. He noted clear improvement following the biological mesh reconstruction and reported no further wound problems at one year.

Case 2: The patient said the long course of complications, and the vascular emergency in particular, had been very difficult. After the salvage procedure his symptoms resolved and he returned to his normal daily activities without limitation at one year of follow-up.

## Data Availability

The original contributions presented in the study are included in the article/Supplementary Material, further inquiries can be directed to the corresponding author.
